# A Novel Approach To Predict Glaucomatous Impairment in the Central 10° Visual Field, Excluding the Effect of Cataract

**DOI:** 10.1167/tvst.13.10.35

**Published:** 2024-10-24

**Authors:** Ryo Tomita, Ryo Asaoka, Kazunori Hirasawa, Yuri Fujino, Tetsuro Omura, Tsutomu Inatomi, Akira Obana, Koji M. Nishiguchi, Masaki Tanito

**Affiliations:** 1 Department of Ophthalmology, Nagoya University Graduate School of Medicine, Nagoya, Aichi, Japan; 2 Department of Ophthalmology, Seirei Hamamatsu General Hospital, Hamamatsu, Shizuoka, Japan; 3 Seirei Christopher University, Hamamatsu, Shizuoka, Japan; 4 The Graduate School for the Creation of New Photonics Industries, Hamamatsu, Shizuoka, Japan; 5 Organization for Innovation and Social Collaboration, National University Corporation Shizuoka University, Hamamatsu, Shizuoka, Japan; 6 Department of Ophthalmology, Kitasato University School of Medicine, Sagamihara, Kanagawa, Japan; 7 Department of Ophthalmology, Shimane University Faculty of Medicine, Izumo, Shimane, Japan; 8 Department of Ophthalmology, National Center for Geriatrics and Gerontology, Ohbu, Aichi, Japan; 9 Department of Medical Spectroscopy, Institute for Medical Photonics Research, Preeminent Medical Photonics Education & Research Center, Hamamatsu University School of Medicine, Hamamatsu, Shizuoka, Japan

**Keywords:** glaucoma, cataract, visual field, ganglion cell-Inner plexiform layer, optical coherence tomography

## Abstract

**Purpose:**

Our previous study predicted genuine glaucomatous visual field (VF) impairment in the central 10° VF, excluding the effect of cataract, using visual acuity (VA) and global indexes of VF more accurately than pattern deviation (PD). This study aimed to improve the accuracy by using pointwise total deviation (TD) values with the machine-learning method of random forest model (RFM) and to investigate whether incorporating optical coherence tomography–measured ganglion cell-inner plexiform layer (GCIPL) thickness is useful.

**Methods:**

This retrospective study included 89 eyes with open-angle glaucoma that underwent successful cataract surgery (with or without iStent implantation or ab interno trabeculotomy). Postoperative TD in each of the 68 VF points was predicted using preoperative (1) PD, (2) VA and VF with a linear regression model (LM), and (3) VA and VF with RFM, and averaged as predicted mean TD (mTD_post_). Further prediction was made by incorporating the preoperative GCIPL into the best model.

**Results:**

The mean absolute error (MAE) between the actual and predicted mTD_post_ with RFM (1.25 ± 1.03 dB) was significantly smaller than that with PD (3.20 ± 4.06 dB, *p* < 0.01) and LM (1.42 ± 1.06 dB, *p* < 0.05). The MAEs with the model incorporating GCIPL into RFM (1.24 ± 1.04 dB) and RFM were not significantly different.

**Conclusions:**

Accurate prediction of genuine glaucomatous VF impairment was achieved using pointwise TD with RFM. No merit was observed by incorporating the GCIPL into this model.

**Translational Relevance:**

This pointwise RFM could clinically reduce cataract effect on VF.

## Introduction

Glaucoma is a progressive disease that leads to irreversible visual field (VF) defects.^1^ The Humphrey Field Analyzer (HFA; Carl Zeiss Meditec, Dublin, CA) is commonly used to assess these defects by measuring the VF threshold at multiple test points. The accurate assessment of genuine glaucomatous VF impairment in eyes with both cataract and glaucoma is challenging, because media opacities, such as cataract, lead to diminished VF sensitivity.^2^ In HFA, pattern deviation (PD) is used to estimate genuine glaucomatous VF impairment, minimizing the effect of diffuse sensitivity loss by cataract and other intermediate opacities. Total deviation (TD) value is a result of comparison of each VF point's threshold to an age-matched normal value, whereas PD value is calculated to correct for the generalized depression to highlight localized VF defects.^3^

However, our studies have shown that the estimation of genuine glaucomatous VF impairment by PD can be inaccurate both in the HFA 24-2 and 10-2 tests; it is underestimated in moderate or advanced glaucoma, on the other hand, it is overestimated when cataract is mild.^4^^,^^5^ To overcome this problem, we developed a method to predict mean TD value of postoperative VF (VF_post_), using global indices (mean TD and pattern standard deviation [PSD]) of preoperative VF (VF_pre_) and visual acuity (VA).^5^ The results showed that the prediction was more accurate than that for preoperative PD. However, it still remained a future work to improve prediction accuracy using the pointwise TD value in VF_pre_. For this purpose, ordinal multivariate linear regression may be insufficient because the number of variables is large (68 pointwise TD values). Ganglion cell-inner plexiform layer (GCIPL) thickness, measured by optical coherence tomography (OCT), correlates well with the degree of glaucomatous VF impairment.^6^^–^^10^ Because cataract has a small effect on OCT-measured retinal thickness,^11^^,^^12^ the prediction performance for genuine glaucomatous VF impairment, without the effect of cataract, may be improved by using GCIPL thickness.

In this study, we aimed to determine whether pointwise TD values in VF_post_ can be predicted using pointwise TD values in VF_pre_. In addition, we aimed to ascertain whether incorporating the thickness of the OCT-measured GCIPL measured before surgery further improves prediction performance.

## Methods

### Ethics Approval

This study involving human participants received ethical approval from the Institutional Review Board and the Ethics Committee of Nagoya University Graduate School of Medicine (ID 2021-0477), as well as the Institutional Review Boards of Seirei Hamamatsu General Hospital and Shimane University. The institutional review board exempted this study from informed consent due to the retrospective study design. We published the study protocol on the website and offered participants the opportunity to opt out.

### Participants

Data were retrospectively compiled from 89 eyes of 74 patients diagnosed with primary open-angle glaucoma, who underwent complication-free solo cataract surgery, cataract surgery with iStent trabecular micro-bypass (iStent; Glaukos Corp., San Clemente, CA, USA) implantation, or cataract surgery with ab interno trabeculotomy using the Tanito ab interno microhook (Inami & Co., Ltd., Tokyo, Japan) between April 2018 and July 2022 at Seirei Hamamatsu General Hospital, Nagoya University Hospital, and Shimane University Hospital.

Primary open-angle glaucoma was defined as (1) the presence of typical glaucomatous changes, such as a disk rim notch and retinal nerve fiber layer defect, identified by ophthalmoscopy or fundus photography, and (2) gonioscopically wide-open angles of grade 3 or 4 based on the Shaffer classification. All eyes had reproducible glaucomatous VF defects with the HFA 24-2 test meeting the criteria of Anderson and Patella^13^ as follows: (1) a cluster of ≥3 points in the pattern deviation plot within a single hemifield (superior or inferior) with *P* < 0.05, one of which should be *P* < 0.01, (2) Glaucoma Hemifield Test results above normal limits or (3) abnormal PSD with *P* < 0.05. Exclusion criteria were as follows: (1) previous ophthalmic surgery, (2) any ocular disease besides cataract and glaucoma affecting visual function, and (3) history of cerebral or other ocular pathologies that could affect VF results.

### Surgery

Cataract surgery was performed in 89 eyes, including three solo cataract surgeries, 27 cases of cataract surgery in conjunction with iStent implantation, and 59 cases of cataract surgery plus ab interno trabeculotomy using the Tanito microhook. Cataract surgery was performed as standard phacoemulsification with intraocular lens (IOL) implantation under topical anesthesia using a clear corneal incision. In the combined surgeries, a first- or second-generation iStent inject W was implanted after cataract surgery, or ab interno trabeculotomy was performed using the Tanito microhook. Postoperative complications were closely monitored through slit-lamp and ophthalmoscopic examinations, as well as intraocular pressure measurements. Cases with complications that could affect VF results were excluded, such as hyphema, intraocular pressure spike of 30 mm Hg or increase of >10 mm Hg from preoperative intraocular pressure, macular edema, vitreous hemorrhage, or endophthalmitis.

### VF Testing

VF_pre_ was measured within six months before surgery, and VF_post_ was measured within 12 months from VF_pre_, using the standard 10-2 program of the Swedish interactive threshold algorithm. Only reliable VF measurements were included in the analysis––defined as fixation loss <20%, false-negative error <33%, and false-positive error <33%.

### OCT Imaging

OCT imaging was conducted within six months prior to surgery using an RS-3000 instrument (NIDEK Co., Ltd., Aichi, Japan). Before imaging, the pupil was dilated using a combination of 0.5% tropicamide and 0.5% phenylephrine hydrochloride eye drops (Mydrin-P; Santen Pharmaceutical Co., Ltd., Osaka, Japan). Raster scans were obtained, encompassing a 9 × 9 mm range, and exported as 65,536 (128 × 512) pixels of GCIPL thickness. Data with a signal strength index >6 were included.

The thickness of the GCIPL at each pixel within 10° from the center of the image was assigned to the nearest test point among the 68 points in the 10-2 VF. The assignment was processed using the Littman correction formula to adjust for axial length,^14^^,^^15^ and Dacey's formula was used to convert retinal arcs from millimetres to degrees.^16^ Drasdo's model was adopted to calculate the displacement of each OCT point.^17^ Data obtained from the left eye were mirrored to the right eye.

### Models for Predicting Glaucomatous VF Impairment

Mean postoperative TD (mTD_post_) was considered to represent genuine glaucomatous VF impairment because the effects of cataract were removed by surgery, whereas mean preoperative TD (mTD_pre_) was considered to be a result of both cataract and glaucoma. Difference between the means of total deviation values in the pre- and postoperative visual fields (ΔmTD), defined as the difference between mTD_post_ and mTD_pre_, was considered to represent the effect of cataract.

First, the accuracy of preoperative PD in predicting postoperative TD (TD_post_) was investigated at each test point. Subsequently, the following methods were used to predict TD_post_ at each VF point:

(1) The first prediction method to predict TD_post_ was the linear regression model (LM), inherited from our previous report.^4^^,^^5^ TD_post_ was predicted at each VF point using multivariate linear regression with six variables of first- and second-order preoperative PSD, VA, and TD (TD_pre_). Preoperative VA was represented as the logarithm of the minimum angle of resolution (logMAR) score. The optimal model for the TD_post_ at each VF point was identified using the Akaike information criterion (AIC) with correction for model selection. The AIC is a metric used to balance the goodness of fit and complexity of statistical models.^18^ It penalizes models with more parameters to prevent overfitting. AIC with correction adjusts for bias in AIC with small sample sizes.^19^
(2) The second method to predict TD_post_ value was the random forest model (RFM) with preoperative VA and all 68 TD_pre_ values as independent variables. Random forest is a versatile and widely used machine-learning technique for both classification and regression.^20^ It is an ensemble method that combines multiple decision trees, resulting in a model that is more robust and less susceptible to overfitting than the single decision tree method.

Further predictions were made by incorporating the GCIPL into the model with the lowest prediction error. If LM was the best model, GCIPL thickness assigned to each point of 10-2 VF was added to LM; if RFM was the best model, GCIPL thickness assigned to all 68 points was added to RFM (RFM_OCT_pre_). Additionally, to evaluate the prediction accuracy of OCT, a random forest model with GCIPL thickness at all 68 VF points and VA without any VF data was also developed and the prediction error was calculated in the same way.

The prediction performances of all prediction methods were compared using the leave-one-out cross-validation, in which one or both eyes of a patient were used as test data, whereas the remaining eyes were used as training data. Once the optimal formula was identified using the training data, it was applied to the test data to calculate the predicted TD_post_. This calculation was performed for all the VF points. All processes were iterated 74 times until all participants were used once as the validation data.

The predicted mTD_post_ was the average of all predicted TD_post_ values at 68 points of 10-2 VF in the eye. The prediction error was calculated by subtracting the predicted value from the actual value.

### Statistical Analyses To Compare Variables

The mTD_pre_ and other preoperative values of VA, mean deviation (MD), mean PD_,_ and PSD were compared with the corresponding mTD_post_ and other postoperative values of VA, MD, mean PD, and PSD, respectively, using the linear mixed model. In this study, the measurements were nested within the participants, making them interdependent. Given that the eyes of the same patient were included in some cases, linear mixed models were used for all the comparisons. The linear mixed model accounts for the hierarchical structure of the data and within-subject measurements to minimize potential bias due to the nested nature of the data. The mean absolute error (MAE) of the prediction for mTD_post_ was compared between the three models with the linear mixed model. The Bonferroni's correction was used to adjust for multiple comparisons.

The relationship between the error of each model and mTD_pre_, mTD_post,_ and ΔmTD was investigated. All statistical analyses were conducted using the R programming language version 4.2.2 (The R Foundation for Statistical Computing, Vienna, Austria).

## Results

The demographic information is presented in the Table. A statistically significant difference was observed between preoperative VA and postoperative VA logMAR scores (0.13 ± 0.20 and 0.01 ± 0.13, respectively; *P* < 0.01, linear mixed model). Significant differences were observed between preoperative MD and postoperative MD (−10.22 ± 8.18 dB and −9.76 ± 8.52 dB, respectively; *P* = 0.01, linear mixed model) and between mTD_pre_ and mTD_post_ (−10.30 ± 8.21 and −9.85 ± 8.53 dB, respectively; *P* = 0.02, linear mixed model). No significant differences were observed between preoperative mean PD and postoperative mean PD (−7.30 ± 5.47 and −7.65 ± 5.65 dB, respectively; *P* = 0.05, linear mixed model) or preoperative PSD and postoperative PSD (7.87 ± 4.96 and 8.26 ± 5.05 dB, respectively; *P* = 0.13, linear mixed model).

**Table.** Subjects’  Demographics


Gender	
Male	34
Female	40
Eye	
Right	37
Left	52
Age, year (mean ± SD)	70.38 ± 7.88
Surgery	
Cataract	3
Cataract plus micro-bypass	27
Cataract plus ab interno trabeculotomy	59
Axial length, mm (mean ± SD)	24.79 ± 1.63
Preoperative visual acuity, LogMar (mean ± SD)	0.13 ± 0.20
Postoperative visual acuity, LogMar (mean ± SD)	0.01 ± 0.13^*^
Preoperative MD, dB (mean ± SD)	−10.22 ± 8.18
Postoperative MD, dB (mean ± SD)	−9.76 ± 8.52^*^
mTD_pre_, dB (mean ± SD)	−10.30 ± 8.21
mTD_post_, dB (mean ± SD)	−9.85 ± 8.53^*^
Preoperative mean PD, dB (mean ± SD)	−7.30 ± 5.47
Postoperative mean PD, dB (mean ± SD)	−7.65 ± 5.65
Preoperative PSD, dB (mean ± SD)	7.87 ± 4.96
Postoperative PSD, dB (mean ± SD)	8.26 ± 5.05

LogMar, logarithm of the minimum angle of resolution; SD, standard deviation.

* Significantly different from the corresponding preoperative value (*P* < 0.05, linear mixed model).

Differences between preoperative mean PD (i.e., predicted mTD_post_ by PD [−7.30 ± 5.47] and actual mTD_post_, which is defined as representing genuine glaucomatous VF impairment [−9.85 ± 8.53; *P* < 0.01, linear mixed model]) were significant, whereas predicted mTD_post_ by LM (−9.86 ± 7.97 dB), RFM (−9.79 ± 8.23 dB), and RFM_OCT_pre_ (−9.78 ± 8.18 dB) were not significantly different from actual mTD_post_ (*P* = 0.98, *P* = 0.74, and *P* = 0.72, respectively; linear mixed model).

**Figure 1.**
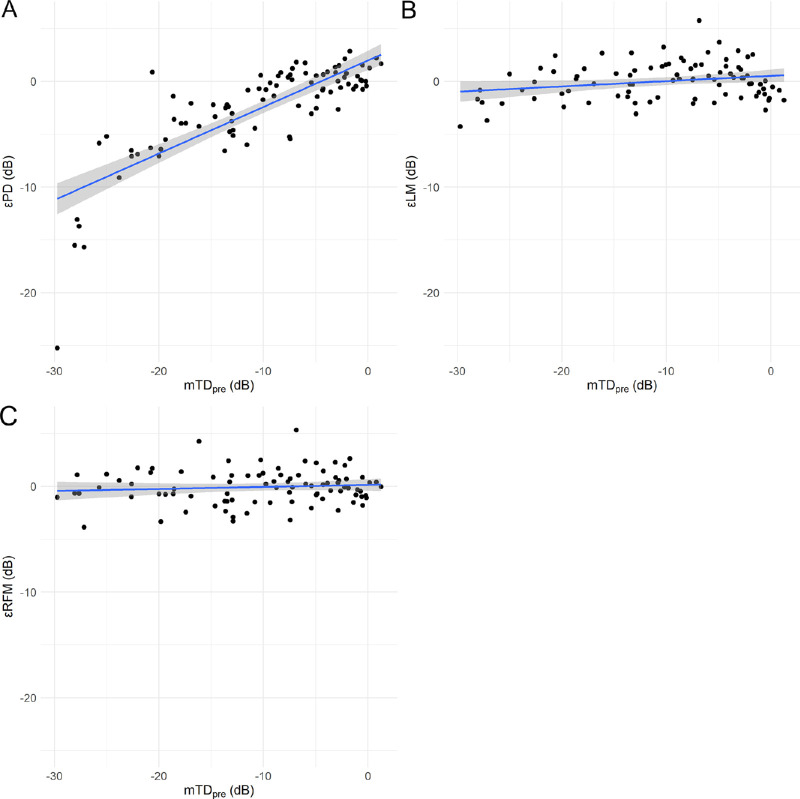
Relationship between mTD_pre_ and εPD, εLM, and εRFM. Correlations between mTD_pre_ and (**A**) εPD, (**B**) εLM, and (**C**) εRFM. The εPD and εLM increased significantly with increasing mTD_pre_.

Figures 1 ^2^ to 3 show the correlations between the errors yielded by the three models (εPD, prediction error of linear regression model (εLM), and prediction error of random forest model with preoperative visual field and visual acuity [εRFM]) and the values of mTD_pre,_ mTD_post,_ and ΔmTD, which is defined as representing the effect of cataract.

**Figure 2.**
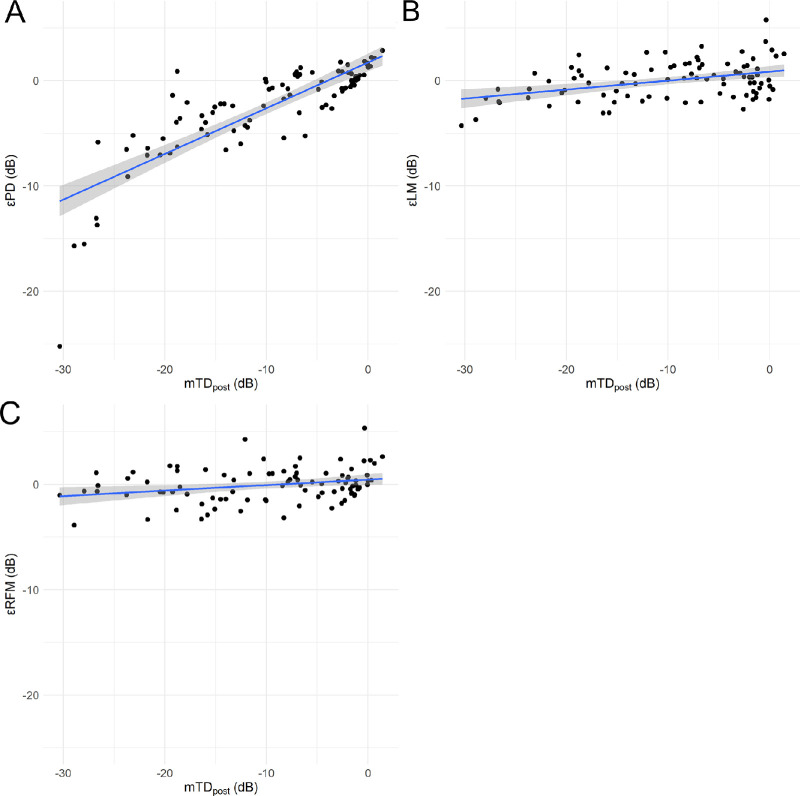
Relationship between mTD_post_ and εPD, εLM, and εRFM. Correlations between mTD_post_ and (**A**) εPD, (**B**) εLM, and (**C**) εRFM. The εPD, εLM, and εRFM increased significantly with increasing mTD_post_.

**Figure 3.**
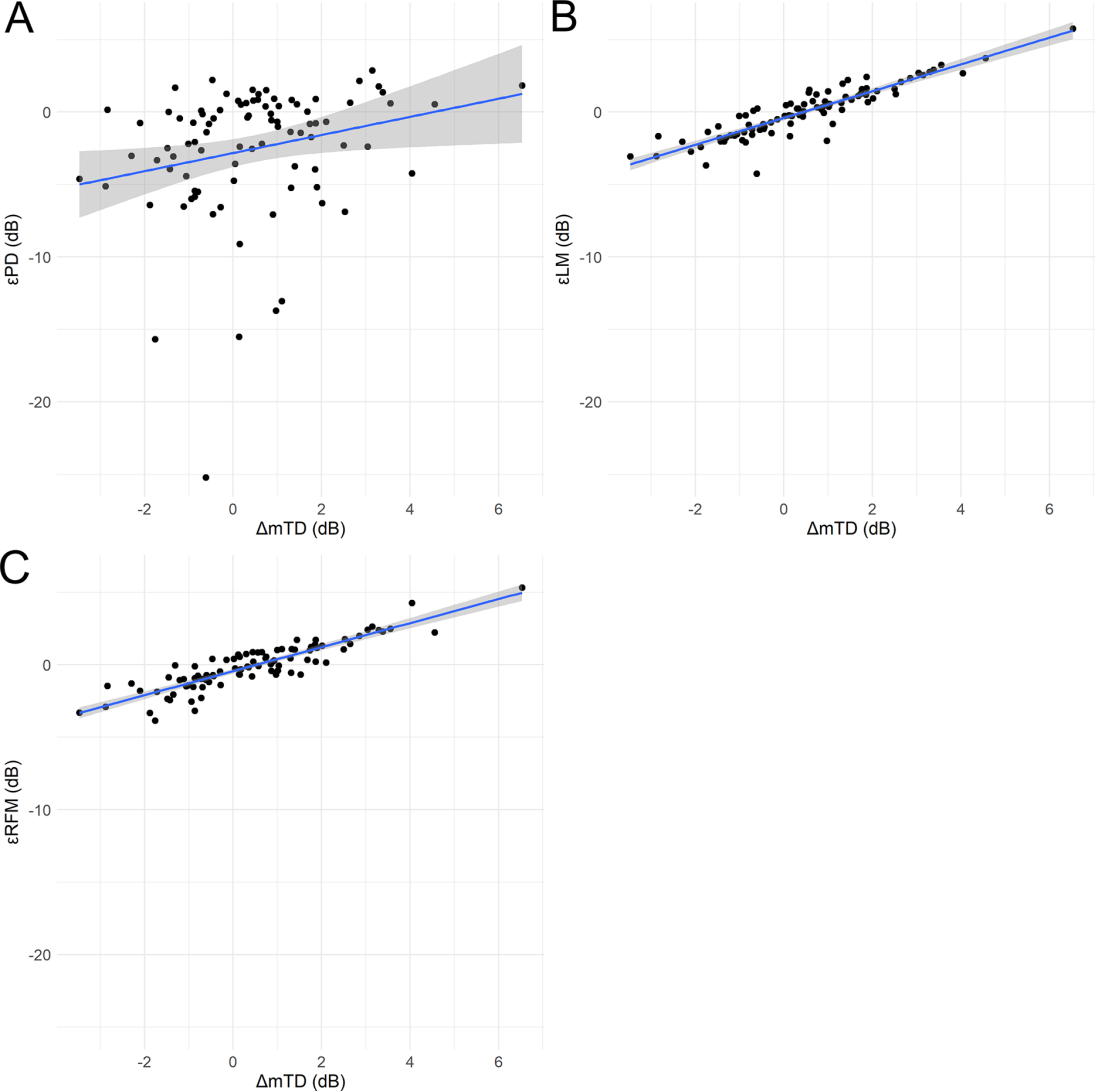
Relationship between ΔmTD and εPD, εLM, and εRFM. Correlations between ΔmTD and (**A**) εPD, (**B**) εLM, and (**C**) εRFM. The εPD, εLM, and εRFM increased significantly with increasing ΔmTD.

• εPD and εLM significantly increased with increasing mTD_pre_: εPD = 1.99 + 0.44 × mTD_pre_ (standard error [SE] = 0.04, *P* < 0.01, linear mixed model) (Fig. 1A); εLM = 0.51 + 0.05 × mTD_pre_ (SE = 0.02, *P* = 0.03, linear mixed model) (Fig. 1B) and εRFM = 0.14 + 0.02 × mTD_pre_ (SE = 0.02, *P* = 0.36, linear mixed model) (Fig. 1C).
• εPD, εLM, and εRFM significantly increased with increasing mTD_post_: εPD = 1.80 + 0.44 × mTD_post_ (SE = 0.03, *P* < 0.01, linear mixed model) (Fig. 2A); εLM = 0.84 + 0.09 × mTDpost (SE = 0.02, *P* < 0.01, linear mixed model) (Fig. 2B), and εRFM = 0.46 + 0.05 × mTDpost (SE = 0.02, *P* < 0.01, linear mixed model) (Fig. 2C).
• εPD, εLM, and εRFM significantly increased with increase in ΔmTD: εPD = −2.78 + 0.73 × ΔmTD (SE = 0.26; *P* < 0.01, linear mixed model) (Fig. 3A); εLM = −0.40 + 0.94 × ΔmTD (SE = 0.04; p < 0.01, linear mixed model) (Fig. 3B) and εRFM = −0.43 + 0.84 × ΔmTD (SE = 0.04; p < 0.01, linear mixed model) (Fig. 3C).

**Figure 4.**
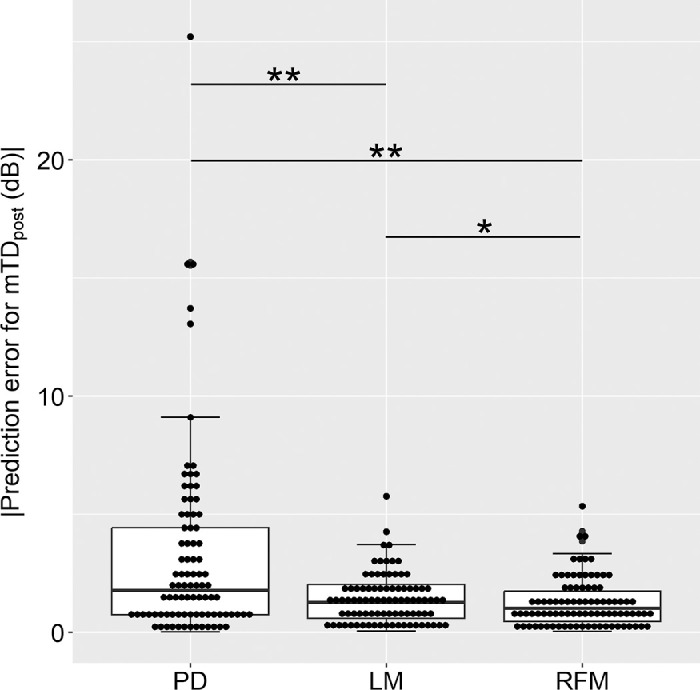
Comparison of absolute prediction errors for PD, LM, and RFM. The MAE of LM (1.42 ± 1.06 dB) was significantly smaller than that of PD (3.20 ± 4.06 dB, *P* < 0.01, linear mixed model adjusted for multiple comparisons using Bonferroni's method). In addition, the MAE of RFM (1.25 ± 1.03 dB) was significantly smaller than that of LM (*P* = 0.03) and PD (*P* < 0.01, linear mixed model adjusted for multiple comparisons using Bonferroni's method). **P < 0.01, *P < 0.05.

The MAE was 3.20 ± 4.06 dB with PD, 1.42 ± 1.06 dB with LM and 1.25 ± 1.03 dB with RFM. The MAE of LM was significantly smaller than those of PD (*P* < 0.01, linear mixed model adjusted for multiple comparisons using Bonferroni's method; Fig. 4). The MAE of RFM was significantly smaller than that of LM and PD (*P* = 0.03 and *P* < 0.01, linear mixed model adjusted for multiple comparisons using Bonferroni's method; Fig. 4). The MAE (1.24 ± 1.04 dB) of RFM_OCT_pre_ was not significantly different from that of RFM (*P* = 0.61). The MAE of the random forest model with GCIPL thickness at all 68 VF points and VA without any VF data was 5.63 ± 4.31 dB.

## Discussion

This study assessed the usefulness of preoperative PD and other prediction models to predict glaucomatous VF (HFA 10-2 test) impairment after cataract surgery in eyes with cataract and glaucoma. As expected, TD values changed significantly after cataract surgery, suggesting the need for a method to predict postoperative TD values before surgery. As indicated in our earlier study, the prediction with PD resulted in a considerably large MAE (3.20 dB) and tended to underestimate glaucomatous VF impairment in eyes with moderate to advanced glaucoma.^5^ Consistently, in this study, the prediction with LM had a significantly smaller MAE than that with PD; however, the prediction with RFM further improved the prediction accuracy.

Of the five eyes with an absolute prediction error larger than 10 dB with PD, the difference between the preoperative mean PD and mTD_pre_ was larger than 10 dB in four eyes. There was no eye with a difference of more than 10 dB in other eyes. This suggests that with a careful consideration is needed when a large difference is observed between preoperative mean PD and mTD_pre_. The difference in the prediction errors with PD (3.2 dB in average) and RFM (1.25 dB in average) was thought to be clinically meaningful, because it is identical or larger than the test–retest reproducibility of the 10-2 VF test, which has been reported as between 0.83 and 2.00 dB.^21^^–^^23^

We conducted this study assuming that it would be useful to use the information from all test points. A weakness of the LM is that it could not handle variables when its number is large, such as 69 in this study. Moreover, pointwise visual sensitivities of VF were closely intercorrelated in glaucoma, which was problematic for linear regression.^24^^–^^26^ Compared to LM, RFM could handle independent variables, irrespective of the large number of variables. In addition, this method could address the intercorrelation of multiple explanatory variables.^27^^,^^28^ Therefore a significantly better prediction was made by RFM than by PD and LM in this study. This prediction with RFM was notably more accurate than that with PD, suggesting its potential to replace PD in clinical settings. However, the prediction error of RFM was significantly affected by the degree of cataract similarly to PD and LM. This issue should be resolved in future studies by using other methods and techniques.

In this study, we investigated whether incorporating OCT-measured GCIPL thickness contributes to improving prediction performance. However, no improvement was observed. One possible reason is that the prediction error with RFM is considerably tight and may have already reached a plateau. Consistently, the test–retest reproducibility of the 10-2 VF test is between 0.83 and 2.00 dB,^21^^–^^23^ which is comparable with the MAE for RFM. Moreover, the prediction error with the random forest model using GCIPL thickness at all 68 VF points and VA (VF data is not used) was much larger compared to the prediction error with RFM (5.63 vs. 1.25 dB), suggesting that the prediction only with structural data was not useful. Furthermore, although only OCT images with better quality were used in this study, it is possible that the improvement of prediction accuracy by incorporating GCIPL was not achieved due to the effect of cataract on the measurements of OCT.^11^^,^^12^ There is a floor effect in the structure–function relationship in glaucoma,^29^^,^^30^ and, hence, the usefulness of GCIPL thickness was probably limited in eyes with advanced glaucomatous damage. In contrast, such a floor effect may not be associated with OCT angiography (OCTA). Moreover, the benefit of using OCTA alone^31^ or OCTA combined with OCT^32^^–^^34^ was reported to improve structure–function relationship. Hence, OCTA may be advantageous, especially in predicting sensitivity at severe VF points that reach the floor. Further studies should be conducted to determine whether the simultaneous use of OCT and OCTA is effective.

This study has several limitations. First, the study cohort included eyes that underwent combined procedures with iStent insertion or trabeculotomy and not solo cataract surgery alone. However, this effect would be marginal because patients with complications such as postoperative intraocular pressure >30 mm Hg and hyphema were carefully excluded. Consistently, no significant differences were observed in the three types of prediction errors (PD, LM, and RFM) across surgery types (data not shown). Second, this study assumed that the TD_post_ indicates genuine glaucomatous impairment and should match the TD_pre_. However, the variability of VF may impact this assumption,^22^^,^^35^ because VF_pre_ and VF_post_ were measured only once.^36^ Therefore further studies using protocols with multiple VF assessments are warranted. Third, there is a period of up to one year between VF_pre_ and VF_post_. The glaucomatous VF impairment may have progressed during the period. Moreover, the VF progression may have been caused by surgery. Fourth, the eyes with IOL may have poorer VF test results at peripheral points compared to the phakic eyes.^37^ However, this effect is significant outside 9° peripheral from the center and may have only a negligible effect in the central 10° VF in the current study. Finally, the relatively small sample size (74 patients, 89 eyes) indicated the need for larger studies to confirm our findings to validate the generalizability and clinical applicability of the current results.

In conclusion, we propose a novel approach to predict glaucomatous VF impairment, excluding the effect of cataract, using pointwise TD values with RFM, which yielded a better prediction than PD. Incorporating OCT-measured GCIPL into this model did not improve the prediction accuracy. These insights have resulted in an updated method for estimating glaucomatous VF impairment in the presence of cataract, which would enhance clinical decision-making and patient management.
